# Supplementary material for: Comparison of alcohol consumption and tobacco use among Korean adolescents before and during the COVID-19 pandemic
Source: PLoS One. 2023 Mar 23;18(3):e0283462. doi: 10.1371/journal.pone.0283462 (PMC10035916; doi:10.1371/journal.pone.0283462)
Supplement: S1 Table — (DOCX) [file pone.0283462.s001.docx]

| **Table 4. Logistic Regression Results on Alcohol Use for 2019 vs 2020, 2019 vs 2021 Participants** | | | | | | | |
| --- | --- | --- | --- | --- | --- | --- | --- |
| **Variables** | **Current Alcohol Use** | | | | | | |
|  |  | **Adjusted OR** | **95% CI** | | |  | **P-value** |
| **COVID-19** | |  |  |  |  |  |  |
| 2021 |  | 0.70* | (0.66 | – | 0.73) |  | <.0001 |
| 2020 |  | 0.70* | (0.66 | – | 0.73) |  | <.0001 |
| 2019 |  | 1.00 |  |  |  |  |  |
| **Sex** | |  |  |  |  |  |  |
| Male |  | 0.67* | (0.64 | - | 0.70) |  | <.0001 |
| Female |  | 1.00 |  |  |  |  |  |
| **Level of School** | |  |  |  |  |  |  |
| Middle school | | 2.86* | (2.72 | - | 2.99) |  | <.0001 |
| High school | | 1.00 |  |  |  |  |  |
| **Scholastic performance** | |  |  |  |  |  |  |
| High |  | 2.14* | (1.98 | - | 2.30) |  | <.0001 |
| Middle |  | 1.71* | (1.60 | - | 1.84) |  | <.0001 |
| Low |  | 1.00 |  |  |  |  |  |
| **Economic level** | |  |  |  |  |  |  |
| High |  | 1.06 | (0.91 | - | 1.24) |  | 0.48 |
| Middle |  | 1.25* | (1.07 | - | 1.45) |  | 0.00 |
| Low |  | 1.00 |  |  |  |  |  |
| **Educational level of father** | |  |  |  |  |  |  |
| College or over | | 1.61* | (1.39 | - | 1.87) |  | <.0001 |
| High school | | 1.17* | (1.01 | - | 1.35) |  | 0.03 |
| Middle school or less | | 1.00 |  |  |  |  |  |
| **Educational level of mother** | |  |  |  |  |  |  |
| College or over | | 1.19 | (0.99 | - | 1.41) |  | 0.06 |
| High school | | 0.89 | (0.75 | - | 1.06) |  | 0.18 |
| Middle school or less | | 1.00 |  |  |  |  |  |
| **Subjective health status** | |  |  |  |  |  |  |
| High |  | 1.10* | (1.02 | - | 1.19) |  | 0.01 |
| Middle |  | 1.13* | (1.04 | - | 1.23) |  | 0.00 |
| Low |  | 1.00 |  |  |  |  |  |
| **Stress level** | |  |  |  |  |  |  |
| Low |  | 1.70* | (1.60 | - | 1.82) |  | <.0001 |
| Middle |  | 1.42* | (1.36 | - | 1.49) |  | <.0001 |
| High |  | 1.00 |  |  |  |  |  |
| **BMI** | |  |  |  |  |  |  |
| Overweight & Obese | | 0.94* | (0.89 | – | 1.00) |  | 0.03 |
| Normal |  | 1.00 |  |  |  |  |  |

Statistically significant was marked as *. Abbreviations: CI, Confidence interval; OR, Odds ratio

| **Table 4. Logistic Regression Results on Smoking for 2019 vs 2020, 2019 vs 2021 Participants** | | | | | | | |
| --- | --- | --- | --- | --- | --- | --- | --- |
| **Variables** | **Current Smoking** | | | | | | |
|  |  | **Adjusted OR ^a^** | **95% CI** | | |  | **P-value** |
| **COVID-19** | |  |  |  |  |  |  |
| 2021 | | 0.66* | (0.61 | – | 0.71) |  | <.0001 |
| 2020 | | 0.66* | (0.61 | – | 0.71) |  | <.0001 |
| 2019 | | 1.00 |  |  |  |  |  |
| **Sex** | |  |  |  |  |  |  |
| Male | | 0.35* | (0.33 | - | 0.38) |  | <.0001 |
| Female |  | 1.00 |  |  |  |  |  |
| **Level of School** | |  |  |  |  |  |  |
| Middle school | | 3.35* | (3.10 | - | 3.63) |  | <.0001 |
| High school | | 1.00 |  |  |  |  |  |
| **Scholastic performance** | |  |  |  |  |  |  |
| High |  | 4.80* | (4.32 | - | 5.33) |  | <.0001 |
| Middle |  | 2.67* | (2.44 | - | 2.92) |  | <.0001 |
| Low |  | 1.00 |  |  |  |  |  |
| **Economic level** | |  |  |  |  |  |  |
| High |  | 1.01 | (0.82 | - | 1.24) |  | 0.94 |
| Middle |  | 1.35* | (1.10 | - | 1.66) |  | 0.00 |
| Low |  | 1.00 |  |  |  |  |  |
| **Educational level of father** | |  |  |  |  |  |  |
| College or over | | 1.17* | (0.93 | - | 1.48) |  | 0.18 |
| High school | | 0.90 | (0.72 | - | 1.14) |  | 0.38 |
| Middle school or less | | 1.00 |  |  |  |  |  |
| **Educational level of mother** | |  |  |  |  |  |  |
| College or over | | 1.48* | (1.15 | - | 1.90) |  | 0.00 |
| High school | | 1.14 | (0.89 | - | 1.46) |  | 0.31 |
| Middle school or less | | 1.00 |  |  |  |  |  |
| **Subjective health status** | |  |  |  |  |  |  |
| High |  | 1.04 | (0.92 | - | 1.17) |  | 0.54 |
| Middle |  | 1.12 | (0.98 | - | 1.27) |  | 0.09 |
| Low |  | 1.00 |  |  |  |  |  |
| **Stress level** | |  |  |  |  |  |  |
| Low |  | 1.86* | (1.60 | - | 2.06) |  | <.0001 |
| Middle |  | 1.65* | (1.53 | - | 1.78) |  | <.0001 |
| High |  | 1.00 |  |  |  |  |  |
| **BMI** | |  |  |  |  |  |  |
| Overweight & Obese | | 1.20* | (1.10 | – | 1.31) |  | <.0001 |
| Normal |  | 1.00 |  |  |  |  |  |

Statistically significant was marked as *. Abbreviations: CI, Confidence interval; OR, Odds ratio
