# Supplementary material for: Comparison of alcohol consumption and tobacco use among Korean adolescents before and during the COVID-19 pandemic
Source: PLoS One. 2023 Mar 23;18(3):e0283462. doi: 10.1371/journal.pone.0283462 (PMC10035916; doi:10.1371/journal.pone.0283462)
Supplement: Attachment — Submitted filename: Manuscript_Editors comments.docx [file pone.0283462.s003.docx]

**Comparison of Alcohol Consumption and Tobacco Use Among Korean Adolescents Before and During the COVID-19 Pandemic**

Wonseok Jeong

Department of Public Health, Graduate School, Seoul National University, Seoul, Republic of Korea

*Corresponding author: Wonseok Jeong, B.A in Economics

Department of Public Health Policy, Graduate School, Seoul National University, 1 Gwanak-ro, Gwanak-gu, Seoul 08826, Republic of Korea

Tel: +82-02-880-2740; E-mail: wsjeong22@snu.ac.kr

Number of tables: 4

**Abstract**

*Background:* The COVID-19 pandemic has brought significant changes all around the world, and due to the strict “Social Distancing Plan”, which includes school closures, Korean adolescents especially have experienced unprecedented changes in their lives. Meanwhile, the presence of peers affects the health behaviors of adolescents significantly, and since substance uses have critical impact on the healthy development of adolescents, the change in substance uses among youths due to the pandemic has to be investigated. Therefore, this study aimed to examine how these risk behaviors among Korean adolescents have changed before and during the COVID-19 pandemic.

*Methods:* Data were included from 2019, 2020, and 2021 Korea Youth Risk Behavior Web-based Survey of 87,532 adolescents. The KYRBWS is conducted by a national institution which use a stratified multistage sampling, and the data is statistically reliable and representative of the population.. The odds ratios (ORs) with 95% CIs for current smoking status and current drinking status among 2020 and 2021 participants were compared with those of the 2019 participants using multiple logistic regression analysis.

*Results:* The degree of current smoking status was lower in 2020 and 2021 participants than in the 2019 participants (2020: OR = 0.66, 95% CI = 0.61–0.71; 2021: OR = 0.66, 95% CI = 0.61–0.71). On the same token, current drinking status was also lower in the participants during the pandemic than those before the pandemic (2020: OR = 0.70, 95% CI = 0.66–0.73; 2021: OR = 0.70, 95% CI = 0.66–0.73).

*Conclusion:* This study found that alcohol consumption and tobacco use had inverse associations with the COVID-19 pandemic among Korean adolescents.

Keywords: Covid-19 Pandemic, health behaviors, adolescents, drinking, smoking, substance use

1. **Introduction**

COVID-19 was first recognized in December 2019 in Wuhan, China, and due to its high contagiousness, as of March 18, 2020, more than 218,000 infected patients and 8,900 deaths had been reported, and the virus had reached 173 countries [1]. As of 31 March 2020, a total of 9786 confirmed cases with COVID-19 have been reported in South Korea as well [2]. Subsequently, on 11 March 2020, the World Health Organization announced that COVID-19 was a global pandemic, and in order to control the outbreak and minimize its spread, the South Korean government enforced a strict “Social Distancing Plan” which includes contact tracing, quarantine, social distancing and school closures.

Meanwhile, adolescence is often associated with an increased need for social connection and peer acceptance, and a heightened sensitivity to peer influence [3]. Indeed, the presence of friends increases the likelihood that adolescents will take certain risks; having friends who smoke or drink is one of the biggest predictors of adolescent engagement in these behaviors [4]. Furthermore, according to the pervious study, peer differentiation strongly influences psychosocial maturity of adolescents, which can also eventually lead to problematic behaviors [5]. These factors refer that social distancing rules and the school closures due to the COVID-19 pandemic may have especially affected the risk and health behaviors of young people, more than did to the other age groups.

Furthermore, risk behaviors among adolescents are extremely maleficent since such behaviors often extend into adulthood, rendering them vulnerable in adulthood to preventable morbidities and mortalities [4]. For instance, adolescents who drink alcohol are more likely to have alcohol-related disorders, mental health problems, and chronic diseases in adulthood, while smoking in adolescents is positively associated with nicotine-dependent, cancer and cardiovascular diseases in adulthood [6].

As such, the Korean government have enforced a “stay-at-home” orders due to the COVID-19 outbreak, when youths’ risk behaviors are highly influenced by the peers. Thus, behavior changes of adolescents highly likely have occurred and youths’ risk behaviors require special attentions. Prior studies, however, only targeted on adults or focused on different matters of adolescents, and have not yet scrutinized on the patterns of substance use changed of Korean adolescents due to the pandemic. This led us to compare the alcohol consumption and tobacco use before and during COVID-19 pandemic among South Korean adolescents.

2. **Materials and methods**

**Data and study participants**

Data for this study were taken from a sample of the 2019, 2020, and 2021 Korea Youth Risk Behavior Web-based Survey (KYRBWS), which collects information on the health-risk behaviors in Korean students in the seventh to the twelfth grades. The KYRBWS is conducted by a national institution, the Korea Centers for Disease Control and Prevention, based on stratified two-stage random cluster sampling, and therefore, the data gained from it is statistically reliable and representative in comparison to surveys performed by private institutions. The KYRBWS was approved by the KCDC Institutional Review Board (2014-06EXP-02-P-A) in 2014. From 2015, the ethics approval for the KYRBWS was waived by the KCDC Institutional Review Board under the Bioethics & Safety Act and opened to the public. Also, the written informed consent was obtained from each students’ parents for the survey [7]. Lastly, as the data is publicly available, no approval related to the ethics of research was needed. All data is available from https://www.kdca.go.kr/yhs/home.jsp with the permission of the reasonable request.

Of the 167,099 individuals who participated in the surveys, we first excluded those aged >19 years (n=166,590). Nobody was excluded from the dependent variables: drinking status and smoking status. Next, among 166,590, 87,532 remained due to the missing data from the variable ‘educational level of father and mother.’ Finally, there was no missing data in other covariates such as economic level, subjective health status, stress level, and BMI, leading the remaining 87,532 as the final study sample size.

**Variables**

The primary independent variable was the COVID-19 pandemic. Since the first patient with COVID-19 in South Korea was diagnosed on February 19, 2020, the study compared alcohol consumption and tobacco use between 2019 (before), 2020 and 2021 (during) participants [8].

The demographic characteristics included in the study were participants’ age (14−16 (Middle School), 17−19 (High School)) and gender. Socioeconomic factors included participants’ scholastic performance (high, middle, and low), subjective economic level (low, middle, and high) and education level of parents (middle school or less, high school, and college or over). The health-related characteristics included the participants’ self-reported health status (high, middle, and low), stress level (high, middle, and low) and body mass index (normal, and overweight & obese). Participants with >=25 BMI were categorized as overweight & obese.

Alcohol consumption and tobacco use were included as the main dependent variables in this study. Participants who smoked more than one conventional cigarette within a month was categorized as smokers and those who have never smoked or have not smoked a single conventional cigarette within a month were categorized as non-smokers. Similarly, participants who drank at least one glass of any type of alcohol within a month were categorized as drinker while those who have never drank or have not drunk a single glass of alcohol within a month were categorized as non-drinkers. All answers were based on self-reported measures.

**Statistical Analysis**

Chi-squared tests were conducted to assess the association of demographic and socioeconomic characteristicsof the study population with their substance use. A multiple logistic regression analysis was performed to compare the alcohol consumption and tobacco use before and after the COVID-19 pandemic after accounting for potential confounding variables, including demographic, socioeconomic, and health-related characteristics. Results are reported as odds ratios (OR) with a 95% confidence interval (CI). Differences were considered statistically significant with a *p*-value of <0.05. All data analyses were conducted using SAS 9.4 software (version 9.4; SAS Institute Inc., Cary, NC, USA).

3. **Results**

A total of 167,099 participants were included. After all exclusions, data from 87,532 participants were analyzed. Table 1 presents the general characteristics of the study population; 27,205 youths in the 2019 were compared with 30,366 youths in the 2020 and 29,961 youths in the 2021. In 2019, 5.6% and 14.2% of the participants smoked conventional cigarettes and drank alcohol, respectively. In 2020, the rates have decreased by 3.8% and 10.2%, and it continued to reduce until 3.6% and 10.0% in 2021. The 2019 participants had the lower severe stress level (39.9%) than that of 2020 (33.6%) and 2021 (38.9% ) participants. Also, 2019 youths had the lowest overweight and obese rate of 14.7% compared to 15.9% and 16.7% from 2020 and 2021 participants.

| **Table 1. General characteristics of Participants (n=87532)** | | | | | | | |  |  |  |  |
| --- | --- | --- | --- | --- | --- | --- | --- | --- | --- | --- | --- |
| **Variables** | | **Total** | |  | **Participants, No (%)** | | | | | |  |
|  |  |  |  |  | **2019** | | **2020** | | **2021** | | **P-value** |
|  |  | 87,532 | (100.0) |  | 27,205 | (31.1) | 30,366 | (34.7) | 29,961 | (34.2) |  |
| **Age (school)** | |  |  |  |  |  |  |  |  |  | < 0.0001 |
| Middle school | | 46,359 | (53.0) |  | 13,979 | (30.2) | 15,900 | (34.3) | 16,480 | (35.5) |  |
| High school | | 41,173 | (47.0) |  | 13,226 | (32.1) | 14,466 | (35.1) | 13,481 | (32.7) |  |
| **Sex** |  |  |  |  |  |  |  |  |  |  | 0.0455 |
| Male | | 40,404 | (46.2) |  | 12,409 | (30.7) | 14,165 | (35.1) | 13,830 | (34.2) |  |
| Female | | 47,128 | (53.8) |  | 14,796 | (31.4) | 16,201 | (34.4) | 16,131 | (34.2) |  |
| **Scholastic performance** | | | |  |  |  |  |  |  |  | 0.0022 |
| High | | 37,722 | (43.1) |  | 11,908 | (31.6) | 12,831 | (34.0) | 12,983 | (34.4) |  |
| Middle | | 43,428 | (49.6) |  | 13,321 | (30.7) | 15,253 | (35.1) | 14,854 | (34.2) |  |
| Low | | 6,382 | (7.3) |  | 1,976 | (31.0) | 2,282 | (35.8) | 2,124 | (33.3) |  |
| **Economic level of family** | | | |  |  |  |  |  |  |  | 0.0057 |
| High | | 38,428 | (43.9) |  | 11,902 | (31.0) | 13,224 | (34.4) | 13,302 | (34.6) |  |
| Middle | | 47,927 | (54.8) |  | 14,912 | (31.1) | 16,705 | (34.9) | 16,310 | (34.0) |  |
| Low | | 1,177 | (1.3) |  | 391 | (33.2) | 437 | (37.1) | 349 | (29.7) |  |
| **Educational level of father** | | | |  |  |  |  |  |  |  | < 0.0001 |
| College or over | | 61,739 | (70.5) |  | 18,783 | (30.4) | 21,293 | (34.5) | 21,663 | (35.1) |  |
| High school | | 24,323 | (27.8) |  | 7,929 | (32.6) | 8,551 | (35.2) | 7,843 | (32.2) |  |
| Middle school or less | | 1,470 | (1.7) |  | 493 | (33.5) | 522 | (35.5) | 455 | (31.0) |  |
| **Educational level of mother** | | | |  |  |  |  |  |  |  | < 0.0001 |
| College or over | | 58,818 | (67.2) |  | 17,810 | (30.3) | 20,242 | (34.4) | 20,766 | (35.3) |  |
| High school | | 27,589 | (31.5) |  | 9,000 | (32.6) | 9,744 | (35.3) | 8,845 | (32.1) |  |
| Middle school or less | | 1,125 | (1.3) |  | 395 | (35.1) | 380 | (33.8) | 350 | (31.1) |  |
| **Subjective health status** | | | |  |  |  |  |  |  |  | < 0.0001 |
| High | | 60,468 | (69.1) |  | 19,233 | (31.8) | 21,537 | (35.6) | 19,698 | (32.6) |  |
| Middle | | 20,044 | (22.9) |  | 5,903 | (29.5) | 6,586 | (32.9) | 7,555 | (37.7) |  |
| Low | | 7,020 | (8.0) |  | 2,069 | (29.5) | 2,243 | (32.0) | 2,708 | (38.6) |  |
| **Stress level** | |  |  |  |  |  |  |  |  |  | < 0.0001 |
| High | | 17,227 | (19.7) |  | 5,107 | (29.6) | 6,536 | (37.9) | 5,584 | (32.4) |  |
| Middle | | 37,574 | (42.9) |  | 11,234 | (29.9) | 13,626 | (36.3) | 12,714 | (33.8) |  |
| Low | | 32,731 | (37.4) |  | 10,864 | (33.2) | 10,204 | (31.2) | 11,663 | (35.6) |  |
| **BMI** |  |  |  |  |  |  |  |  |  |  | < 0.0001 |
| Normal | | 73,716 | (84.2) |  | 23,205 | (31.5) | 25,540 | (34.6) | 24,971 | (33.9) |  |
| Overweight & Obese | | 13,816 | (15.8) |  | 4,000 | (29.0) | 4,826 | (34.9) | 4,990 | (36.1) |  |
| **Current smoking status** | | |  |  |  |  |  |  |  |  | < 0.0001 |
| Yes | | 3,768 | (4.3) |  | 1,529 | (40.6) | 1,146 | (30.4) | 1,093 | (29.0) |  |
| No | | 83,764 | (95.7) |  | 25,676 | (30.7) | 29,220 | (34.9) | 28,868 | (34.5) |  |
| **Current drinking status** | | | |  |  |  |  |  |  |  | < 0.0001 |
| Yes | | 9,988 | (11.4) |  | 3,876 | (38.8) | 3,109 | (31.1) | 3,003 | (30.1) |  |
| No | | 77,544 | (88.6) |  | 23,329 | (30.1) | 27,257 | (35.2) | 26,958 | (34.8) |  |

Table 2 shows the association between current smoking status and COVID-19 pandemic among Korean adolescents. Participants in 2020 and 20201 presented with lower odd ratios of current smoking status compared to the participants in 2019. These results were statistically significant (2020: OR = 0.66, 95% CI = 0.61–0.71; 2021: OR = 0.66, 95% CI = 0.61–0.71). Those with low scholastic performance had a higher risk of smoking than those with good or moderate performances (high: OR = 4.80, 95% CI = 4.32–5.33; middle: OR = 2.67, 95% CI = 2.44–2.92). Sex was also related to a lower smoking status (male: OR = 0.354, 95% CI = 0.33–0.38). These results were all statistically significant. Finally, those with severe stress had a higher odd ratios of current smoking status than with low and moderate stresses (low: OR = 1.86, 95% CI = 1.68–2.06; middle: OR= 1.65, 95% CI= 1.53–1.78).

| **Table 2. Logistic Regression Results on Drinking for 2019 vs 2020, 2019 vs 2021 Participants** | | | | | | | |
| --- | --- | --- | --- | --- | --- | --- | --- |
| **Variables** | **Current Drinking** | | | | | | |
|  |  | **Adjusted OR** | **95% CI** | | |  | **P-value** |
| **COVID-19** | |  |  |  |  |  |  |
| 2021 |  | 0.70* | (0.66 | – | 0.73) |  | <.0001 |
| 2020 |  | 0.70* | (0.66 | – | 0.73) |  | <.0001 |
| 2019 |  | 1.00 |  |  |  |  |  |
| **Sex** | |  |  |  |  |  |  |
| Male |  | 0.67* | (0.64 | - | 0.70) |  | <.0001 |
| Female |  | 1.00 |  |  |  |  |  |
| **Age** | |  |  |  |  |  |  |
| Middle school | | 2.86* | (2.72 | - | 2.99) |  | <.0001 |
| High school | | 1.00 |  |  |  |  |  |
| **Scholastic performance** | |  |  |  |  |  |  |
| High |  | 2.14* | (1.98 | - | 2.30) |  | <.0001 |
| Middle |  | 1.71* | (1.60 | - | 1.84) |  | <.0001 |
| Low |  | 1.00 |  |  |  |  |  |
| **Economic level** | |  |  |  |  |  |  |
| High |  | 1.06 | (0.91 | - | 1.24) |  | 0.48 |
| Middle |  | 1.25* | (1.07 | - | 1.45) |  | 0.00 |
| Low |  | 1.00 |  |  |  |  |  |
| **Educational level of father** | |  |  |  |  |  |  |
| College or over | | 1.61* | (1.39 | - | 1.87) |  | <.0001 |
| High school | | 1.17* | (1.01 | - | 1.35) |  | 0.03 |
| Middle school or less | | 1.00 |  |  |  |  |  |
| **Educational level of mother** | |  |  |  |  |  |  |
| College or over | | 1.19 | (0.99 | - | 1.41) |  | 0.06 |
| High school | | 0.89 | (0.75 | - | 1.06) |  | 0.18 |
| Middle school or less | | 1.00 |  |  |  |  |  |
| **Self-reported health status** | |  |  |  |  |  |  |
| High |  | 1.10* | (1.02 | - | 1.19) |  | 0.01 |
| Middle |  | 1.13* | (1.04 | - | 1.23) |  | 0.00 |
| Low |  | 1.00 |  |  |  |  |  |
| **Stress level** | |  |  |  |  |  |  |
| Low |  | 1.70* | (1.60 | - | 1.82) |  | <.0001 |
| Middle |  | 1.42* | (1.36 | - | 1.49) |  | <.0001 |
| High |  | 1.00 |  |  |  |  |  |
| **BMI** | |  |  |  |  |  |  |
| Overweight & Obese | | 0.94* | (0.89 | – | 1.00) |  | 0.03 |
| Normal |  | 1.00 |  |  |  |  |  |

Statistically significant was marked as *. Abbreviations: CI, Confidence interval; OR, Odds ratio

Table 3 shows the association between current drinking status and COVID-19 pandemic among Korean adolescents. 2020 and 2021 participants presented with lower odd ratios of current drinking status compared to the participants in 2019. These results were statistically significant (2020: OR = 0.70, 95% CI = 0.66–0.73; 2021: OR = 0.70, 95% CI = 0.66–0.73). Those with low scholastic performance had a higher risk of drinking alcohol than those with good or moderate performances (high: OR = 2.14, 95% CI = 1.98–2.30; middle: OR = 1.71, 95% CI = 1.60–1.84). Sex was also related to a lower drinking status (male: OR = 0.67, 95% CI = 0.64–0.70). These results were all statistically significant. Finally, those with severe stress had a higher odd ratios of current drinking status than with low and moderate stresses (low: OR = 1.70, 95% CI = 1.60–1.82; middle: OR= 1.42, 95% CI= 1.36–1.49).

| **Table 3. Logistic Regression Results on Smoking for 2019 vs 2020, 2019 vs 2021 Participants** | | | | | | | |
| --- | --- | --- | --- | --- | --- | --- | --- |
| **Variables** | **Current Smoking** | | | | | | |
|  |  | **Adjusted OR** | **95% CI** | | |  | **P-value** |
| **COVID-19** | |  |  |  |  |  |  |
| 2021 | | 0.66* | (0.61 | – | 0.71) |  | <.0001 |
| 2020 | | 0.66* | (0.61 | – | 0.71) |  | <.0001 |
| 2019 | | 1.00 |  |  |  |  |  |
| **Sex** | |  |  |  |  |  |  |
| Male | | 0.35* | (0.33 | - | 0.38) |  | <.0001 |
| Female |  | 1.00 |  |  |  |  |  |
| **Age** | |  |  |  |  |  |  |
| Middle school | | 3.35* | (3.10 | - | 3.63) |  | <.0001 |
| High school | | 1.00 |  |  |  |  |  |
| **Scholastic performance** | |  |  |  |  |  |  |
| High |  | 4.80* | (4.32 | - | 5.33) |  | <.0001 |
| Middle |  | 2.67* | (2.44 | - | 2.92) |  | <.0001 |
| Low |  | 1.00 |  |  |  |  |  |
| **Economic level** | |  |  |  |  |  |  |
| High |  | 1.01 | (0.82 | - | 1.24) |  | 0.94 |
| Middle |  | 1.35* | (1.10 | - | 1.66) |  | 0.00 |
| Low |  | 1.00 |  |  |  |  |  |
| **Educational level of father** | |  |  |  |  |  |  |
| College or over | | 1.17* | (0.93 | - | 1.48) |  | 0.18 |
| High school | | 0.90 | (0.72 | - | 1.14) |  | 0.38 |
| Middle school or less | | 1.00 |  |  |  |  |  |
| **Educational level of mother** | |  |  |  |  |  |  |
| College or over | | 1.48* | (1.15 | - | 1.90) |  | 0.00 |
| High school | | 1.14 | (0.89 | - | 1.46) |  | 0.31 |
| Middle school or less | | 1.00 |  |  |  |  |  |
| **Self-reported health status** | |  |  |  |  |  |  |
| High |  | 1.04 | (0.92 | - | 1.17) |  | 0.54 |
| Middle |  | 1.12 | (0.98 | - | 1.27) |  | 0.09 |
| Low |  | 1.00 |  |  |  |  |  |
| **Stress level** | |  |  |  |  |  |  |
| Low |  | 1.86* | (1.60 | - | 2.06) |  | <.0001 |
| Middle |  | 1.65* | (1.53 | - | 1.78) |  | <.0001 |
| High |  | 1.00 |  |  |  |  |  |
| **BMI** | |  |  |  |  |  |  |
| Overweight & Obese | | 1.20* | (1.10 | – | 1.31) |  | <.0001 |
| Normal |  | 1.00 |  |  |  |  |  |

Statistically significant was marked as *. Abbreviations: CI, Confidence interval; OR, Odds ratio

Table 4 shows the results of a subgroup analysis between two risk behaviors and COVID-19 pandemic, focusing on sex and scholastic performance. Both male and female groups demonstrated higher odds of not smoking and drinking during COVID-19. In addition, according to the scholastic performance, all groups (low, middle, and high) illustrated higher odds of not smoking cigarettes and drinking alcohol in the 2020 and 2021 participants than in the 2019 participants.

| **Table 4. Logistic Regression Results on Substance Uses for 2019 vs 2020, 2019 vs 2021 Participants By Sex and Scholastic Performance** | | | | | | | | | | | |
| --- | --- | --- | --- | --- | --- | --- | --- | --- | --- | --- | --- |
| **Variables** | | **2020** | | | | | **2021** | | | | |
|  |  |  | **^a^ Adjusted OR** | **95% CI** | | |  | **^a^ Adjusted OR** | **95% CI** | | |
| **Sex** | | | |  |  |  |  |  |  |  |  |
| **Men (n=40,404)** | | | |  |  |  |  |  |  |  |  |
| **Current smoking status** | | | |  |  |  |  |  |  |  |  |
| Yes | | | 1.00 |  |  |  |  | 1.00 |  |  |  |
| No | | | 1.59* | (1.44 | – | 1.75) |  | 1.64* | (1.48 | – | 1.81) |
| **Current drinking status** | | | |  |  |  |  |  |  |  |  |
| Yes | |  | 1.00 |  |  |  |  | 1.00 |  |  |  |
| No | |  | 1.46* | (1.36 | – | 1.57) |  | 1.44* | (1.34 | – | 1.55) |
| **Women (n=47,128)** | | | |  |  |  |  |  |  |  |  |
| **Current smoking status** | | | |  |  |  |  |  |  |  |  |
| Yes | |  | 1.00 |  |  |  |  | 1.00 |  |  |  |
| No | |  | 1.47* | (1.36 | – | 1.57) |  | 1.44* | (1.34 | – | 1.55) |
| **Current drinking status** | | | |  |  |  |  |  |  |  |  |
| Yes | |  | 1.00 |  |  |  |  | 1.00 |  |  |  |
| No | |  | 1.41* | (1.31 | – | 1.52) |  | 1.43* | (1.32 | – | 1.54) |
|  |  |  |  |  |  |  |  |  |  |  |  |
| **Scholastic performance** | | | |  |  |  |  |  |  |  |  |
| **High (n=37,722)** | | | |  |  |  |  |  |  |  |  |
| **Current smoking status** | | | |  |  |  |  |  |  |  |  |
| Yes | |  | 1.00 |  |  |  |  | 1.00 |  |  |  |
| No | |  | 1.74* | (1.48 | – | 2.05) |  | 1.80* | (1.53 | – | 2.13) |
| **Current drinking status** | | | |  |  |  |  |  |  |  |  |
| Yes | |  | 1.00 |  |  |  |  | 1.00 |  |  |  |
| No | |  | 1.40* | (1.28 | – | 1.53) |  | 1.36* | (1.24 | – | 1.48) |
| **Middle (n=43,428)** | | | |  |  |  |  |  |  |  |  |
| **Current smoking status** | | | |  |  |  |  |  |  |  |  |
| Yes | |  | 1.00 |  |  |  |  | 1.00 |  |  |  |
| No | |  | 1.53* | (1.37 | – | 1.70) |  | 1.46* | (1.31 | – | 1.62) |
| **Current drinking status** | | | |  |  |  |  |  |  |  |  |
| Yes | |  | 1.00 |  |  |  |  | 1.00 |  |  |  |
| No | |  | 1.51* | (1.41 | – | 1.63) |  | 1.49* | (1.38 | – | 1.60) |
| **Low (n=6,382)** | | | |  |  |  |  |  |  |  |  |
| **Current smoking status** | | | |  |  |  |  |  |  |  |  |
| Yes | |  | 1.00 |  |  |  |  | 1.00 |  |  |  |
| No | |  | 1.27* | (1.07 | – | 1.52) |  | 1.37* | (1.14 | – | 1.65) |
| **Current drinking status** | | | |  |  |  |  |  |  |  |  |
| Yes | |  | 1.00 |  |  |  |  | 1.00 |  |  |  |
| No | |  | 1.25* | (1.08 | – | 1.45) |  | 1.43* | (1.23 | – | 1.66) |

Statistically significant was marked as *. Abbreviations: CI, Confidence interval; OR, Odds ratio

^a^ Adjusted for age, sex, economic level, educational level of father and mother, scholastic performance, subjective health status, stress level and BMI.

4. **Discussion**

There is little empirical evidence on the changes of alcohol and tobacco consumption during the COVID-19 pandemic of Korean teenagers. This study describes the connections between COVID-19 pandemic and its subsequent “stay-at-home” orders and changes in substance uses of Korean adolescents, using demographic, socioeconomic, and health-related variables gained from the 2019, 2020, and 2021 KYRBWS data. There were positive associations with COVID-19 and a statistically significant decreased substance use; adolescents smoked less cigarettes and drank less alcohol compared to how they did before the pandemic. The lower odds of alcohol consumption and cigarettes use were consistent in all subgroups by both sex and scholastic performance.

Social distancing plan and its subsequent school closure due to the COVID-19 outbreak seem to be the reasons for the reduction in the substance uses of teenagers. Adolescence is associated with an increased need for social connection and therefore, presence of friends increases the likelihood of risk behaviors including smoking cigarettes and drinking alcohol [3,4]. Reduced peer pressure and importance of peer relationships from the school closure could have stopped youths from conducting unnecessary risk behaviors. According to the subgroup analysis results of risk behaviors and COVID-19 pandemic in table 4, such peer pressure must have affected adolescents equally regardless of sex and educational performances. In addition, reduced stress due to the stay-at-home orders might also have reduced the substance uses of adolescents. According to the previous study, school closures can alleviate stress in youths by reducing academic burdens and school bullying, while there was a significant association between high perceived stress and cigarette use [9-11]. On the same token, stressful school environment due to South Korea’s educational fever can also place students at risk of alcohol [12]. Lastly, increased family warmth and connectedness due to the social distancing plan might have served as a protective factor against many of the risky behaviors engaged in by adolescents [13]. For example, the prior study presents that in comparison with children who spent more time with their parents, those who spent less time were more inclined to start smoking and try alcohol [14]. Considering that 21.4% of school-aged children found the parent-children discussions to be more satisfying during the school closures, the argument is pretty valid [15].

Yet, the current study’s limitations should be noted. First, the data in this study are based on self-reported measures, and health status measurements might be subject to recall bias. Therefore, caution should be taken when interpreting these results. Also, due to this study’s cross-sectional design, cause and effect, as well as the direction of the relationships observed, could not be determined. Third, cultural aspects could have influenced the impact of the COVID-19 pandemic on smoking and drinking status of Korean adolescents. Lastly, adolescents were considered as current smokers and drinkers if they have smoked or drank at least once within the past thirty days. The exact number of cigarettes smoked and drinks consumed were not involved in the investigation.

Despite these limitations, our study does possess several strengths. The KYRBWS is conducted by a national institution based on random cluster sampling, and therefore, the data gained from it is statistically reliable and representative in comparison to surveys performed by private institutions. Moreover, as this study was conducted for over three years, compared to most recent COVID-19 researches which only include one or two years, the representativeness of the sample was improved upon. Lastly, many covariates, including age, sex, economic level, educational levels of parents, scholastic performance, subjective health status, and BMI, were included to reduce the possible confounding effects.

5. **Conclusions**

Along with the severe health consequences from the pandemic, long-term health consequences from the risk behaviors, especially among adolescents, are significant public health concern. This study found that the substance uses (alcohol and tobacco usage) were reduced among Korean adolescents during the COVID-19 pandemic period regardless of sex and scholastic performance. Despite the decrease, however, we must not belittle the prevalence of risk behaviors among adolescents and the potential effects of the COVID-19 pandemic on adolescents require additional follow-up studies.

Conflicts of interest: None

Sources of support that require acknowledgment: None

**References**

1.	Moradi H, Vaezi A. Lessons learned from Korea: COVID-19 pandemic. Infection Control & Hospital Epidemiology. 2020;41: 873-874

2.	Choi JY. COVID-19 in South Korea. Postgraduate medical journal. 2020;96: 399-402

3.	Andrews JL, Foulkes L, Blakemore S-J. Peer influence in adolescence: Public-health implications for COVID-19. Trends in Cognitive Sciences. 2020;24: 585-587

4.	Loke AY, Mak Y-w. Family process and peer influences on substance use by adolescents. International journal of environmental research and public health. 2013;10: 3868-3885

5.	Gavazzi SM, Goettler DE, Solomon SP, McKenry PC. The impact of family and peer differentiation levels on adolescent psychosocial development and problematic behaviors. Contemporary Family Therapy. 1994;16: 431-448

6.	Fleary SA, Joseph P, Pappagianopoulos JE. Adolescent health literacy and health behaviors: A systematic review. Journal of adolescence. 2018;62: 116-127

7.	Kwon JA, Park E-C, Lee M, Yoo K-B, Park S. Does stress increase the risk of atopic dermatitis in adolescents? results of the Korea Youth Risk Behavior Web-based Survey (KYRBWS-VI). PLoS One. 2013;8: e67890

8.	Kim SY, Kim H-R, Park B, Choi HG. Comparison of stress and suicide-related behaviors among Korean youths before and during the COVID-19 pandemic. JAMA network open. 2021;4: e2136137-e2136137

9.	Tanaka T, Okamoto S. Increase in suicide following an initial decline during the COVID-19 pandemic in Japan. Nature human behaviour. 2021;5: 229-238

10.	Isumi A, Doi S, Yamaoka Y, Takahashi K, Fujiwara T. Do suicide rates in children and adolescents change during school closure in Japan? The acute effect of the first wave of COVID-19 pandemic on child and adolescent mental health. Child abuse & neglect. 2020;110: 104680

11.	Lee A, Lee K-S, Park H. Association of the use of a heated tobacco product with perceived stress, physical activity, and internet use in Korean adolescents: A 2018 national survey. International Journal of Environmental Research and Public Health. 2019;16: 965

12.	Hong JS, Lee NY, Grogan-Kaylor A, Huang H. Alcohol and tobacco use among South Korean adolescents: An ecological review of the literature. Children and Youth Services Review. 2011;33: 1120-1126

13.	Pergamit MR, Huang L, Lane J. The long term impact of adolescent risky behaviors and family environment. Report submitted to Office of the Assistant Secretary for Planning and Evaluation, US Department of Health and Human Services. 2001:

14.	Garmienė A, Žemaitienė N, Zaborskis A. Family time, parental behaviour model and the initiation of smoking and alcohol use by ten-year-old children: an epidemiological study in Kaunas, Lithuania. BMC Public Health. 2006;6: 1-9

15.	Tang S, Xiang M, Cheung T, Xiang Y-T. Mental health and its correlates among children and adolescents during COVID-19 school closure: The importance of parent-child discussion. Journal of affective disorders. 2021;279: 353-360
